# Characterization of the heart transcriptome of the white shark (*Carcharodon carcharias*)

**DOI:** 10.1186/1471-2164-14-697

**Published:** 2013-10-11

**Authors:** Vincent P Richards, Haruo Suzuki, Michael J Stanhope, Mahmood S Shivji

**Affiliations:** 1 Department of Population Medicine and Diagnostic Sciences, College of Veterinary Medicine, Cornell University, Ithaca, NY 14853, USA; 2 Save Our Seas Shark Research Center and Guy Harvey Research Institute, Nova Southeastern University, 8000 North Ocean Drive, Dania Beach, FL 33004, USA; 3 Current address: Graduate School of Science and Engineering, Yamaguchi University, Yoshida 1677-1, Yamaguchi 753-8512, Japan

**Keywords:** White shark, *Carcharodon carcharias*, Heart transcriptome, Microsatellites, Positive selection, Enrichment

## Abstract

**Background:**

The white shark (*Carcharodon carcharias*) is a globally distributed, apex predator possessing physical, physiological, and behavioral traits that have garnered it significant public attention. In addition to interest in the genetic basis of its form and function, as a representative of the oldest extant jawed vertebrate lineage, white sharks are also of conservation concern due to their small population size and threat from overfishing. Despite this, surprisingly little is known about the biology of white sharks, and genomic resources are unavailable. To address this deficit, we combined Roche-454 and Illumina sequencing technologies to characterize the first transciptome of any tissue for this species.

**Results:**

From white shark heart cDNA we generated 665,399 Roche 454 reads (median length 387-bp) that were assembled into 141,626 contigs (mean length 503-bp). We also generated 78,566,588 Illumina reads, which we aligned to the 454 contigs producing 105,014 454/Illumina consensus sequences. To these, we added 3,432 non-singleton 454 contigs. By comparing these sequences to the UniProtKB/Swiss-Prot database we were able to annotate 21,019 translated open reading frames (ORFs) of ≥ 20 amino acids. Of these, 19,277 were additionally assigned Gene Ontology (GO) functional annotations. While acknowledging the limitations of our single tissue transcriptome, Fisher tests showed the white shark transcriptome to be significantly enriched for numerous metabolic GO terms compared to the zebra fish and human transcriptomes, with white shark showing more similarity to human than to zebra fish (i.e. fewer terms were significantly different). We also compared the transcriptome to other available elasmobranch sequences, for signatures of positive selection and identified several genes of putative adaptive significance on the white shark lineage. The white shark transcriptome also contained 8,404 microsatellites (dinucleotide, trinucleotide, or tetranucleotide motifs ≥ five perfect repeats). Detailed characterization of these microsatellites showed that ORFs with trinucleotide repeats, were significantly enriched for transcription regulatory roles and that trinucleotide frequency within ORFs was lower than for a wide range of taxonomic groups including other vertebrates.

**Conclusion:**

The white shark heart transcriptome represents a valuable resource for future elasmobranch functional and comparative genomic studies, as well as for population and other biological studies vital for effective conservation of this globally vulnerable species.

## Background

Cartilaginous fishes (Class Chondrichthyes: sharks, skates, rays, chimaeras) provide a notable example of successful evolutionary perseverance, with a fossil record extending to at least the Lower Devonian over 400 million years ago [1]. Given their extraordinary evolutionary history and basal phylogenetic origin relative to other jawed vertebrates, chondrichthyians have been proposed as an important comparative model for understanding vertebrate genome evolution in general and various specific evolutionary and mechanistic aspects of vertebrate development, physiology and immune function [2-5].

One group of chondrichthyians, the modern sharks (subclass Elasmobranchii), comprise over 500 extant species displaying an impressive diversity of form and function, including a broad spectrum of sizes (e.g. 20-1200 cm as adults), functional morphologies (e.g. fusiform heads to the novel, widened heads of hammerhead sharks), physiology (e.g. ectothermy to regional endothermy), reproduction (e.g. egg laying to live births) and habitat use (marine to freshwater; shallow waters to abyssal depths). Sharks have also become a major target of human exploitation for their fins [6], resulting in widespread concerns that their rapidly declining populations coupled with unique life history characteristics will not permit recovery if ongoing exploitation rates continue. 

Despite representing a major vertebrate lineage of evolutionary uniqueness and ecological and conservation importance, sharks remain the least explored vertebrate group at the genome level. The handful of genome level studies conducted on sharks have already revealed some distinctive features, including the absence of the *HoxC* cluster of developmental pattern genes found in all other non-elasmobranch vertebrate lineages [7], and the presence of a substantial number of expressed sequence tags for which no homologues in other organisms could be identified [8]. These apparent distinctions hint that other genomic novelties are possible in this lineage and await discovery.

The white shark, *Carcharodon carcharias* (Lamnidae), a large apex predator, is one of the highest-profile marine species, capturing extraordinary attention from the public and media. Although it demonstrates a cosmopolitan distribution, the species is believed to have a low abundance throughout its range, leading to international concerns about its conservation (IUCN Red List Category: Vulnerable A2cd+3cd) in the face of known market utilization for its body parts and widespread shark overfishing practices [9-11]. Arguably, the white shark may be a “poster child” for marine, large animal conservation attention. The white shark also possesses some notable physical and physiological characteristics that make it an interesting biological study, including an estimated genome size (C-value = 6.45 pg) nearly twice that of humans, large adult sizes reaching up to ~6 m in length, a thermal regulatory capability uncommon in fishes, a slow reproductive cycle with oophagous embryos, extensive migratory capabilities, and an ability to utilize a wide thermal niche including diving to near 1000 m depths [12-14].

Despite the high public profile of white sharks, their serious conservation needs, and their noteworthy evolutionary and life-history characteristics, this species is still largely uncharacterized at the molecular level, and no genomics resources for it exist. Given the white shark’s rather large genome size, a transcriptome characterization using next-generation sequencing technology provides a tractable entry into providing the first genomic view and genome resource for this remarkable species. However, obtaining white shark tissue is extremely difficult (see Methods), and as a consequence our study was restricted to one tissue type (heart) from one individual. This precluded examination of expression differences among tissue types, and we acknowledge the obvious limitation of a single transcriptome that may not be typical of the species.

Typically, *de-novo* transcriptomes for non-model organisms where no reference genome exists have been obtained using Roche 454 pyrosequencing technology because of the generation of longer sequencing reads e.g. [15-22]. However, recent advances in *de-novo* assembly for shorter Illumina reads are now making this approach a more viable alternative [23]. In addition, some workers have combined both approaches e.g. [15,24], and here we adopt this latter approach for deriving the first transcriptome dataset for the white shark. Specifically, Illumina reads were aligned to 454 contigs to produce a 454/Illumina consensus sequence. By utilizing the strengths of both sequencing technologies, this approach yielded a considerable increase (~20%) in transcriptome annotation when compared to 454 alone. We utilize this sequence dataset to provide a general characterization of the heart transcriptome with regards to gene discovery and annotation, identification and characterization of multiple microsatellite markers, and detection of genes under positive selection.

## Results and discussion

### Assembly

Roche 454 sequencing of the white shark heart cDNA produced 665,399 reads ranging in size from 100-931 bp (median = 387 bp) for a total of 240,894,914 bp. The *de-novo* assembly produced 141,626 contigs (unigenes) ranging in size from 101–12,997 bp, with a mean of 503 bp. The distribution of the number of reads per contig was as follows: 87,500 contigs (62%) = 1 read (singletons), 37,915 contigs (27%) = 2–5 reads, 6,595 contigs (5%) = 6–10 reads, and 9,616 contigs (7%) >10 reads (max = 568). The Illumina HiSeq run produced 78,566,588 100 bp reads. Aligning these data to the 454 contigs produced 105,014 454/Illumina consensus sequences (36,612 454 contigs lacked a consensus sequence). A total of 86,785 (82.6%) of the consensus sequences contained an ORF of 20 amino acids or longer. Of the 454 contigs lacking a 454/Illumina consensus sequence, 3,432 (9.4%) were non-singletons and 2,750 contained an ORF of 20 amino acids or longer. These ORFs were combined with the 86,785 ORFs obtained from the consensus sequences resulting in a total of 89,535 ORFs that were subsequently annotated. For purposes of quantitative evaluation of our combined 454/Illumina approach (e.g. number and length of contigs and number of annotated ORFs), we also processed the 454 data exclusively. Non-singleton 454 contigs (54,126) contained 52,841 ORFs of 20 amino acids or longer (97.6%). The 454 and Illumina derived short read files were deposited in the Sequence Read Archive at NCBI under the study accession number SRP016555. The 454 contigs, 454/Illumina consensus sequences, and 454/Illumina consensus ORFs (89,535) are included as Additional files 1, 2, and 3 respectively.

For a 454 contig, if there were nucleotide sites lacking consensus with Illumina data (possibly due to lack of coverage), the consensus sequence would contain Ns at the relevant positions. This in turn would lead to Xs (unspecified or unknown amino acids) in the subsequent translated ORF. The 86,785 ORFs generated from the 105,014 consensus sequences contained 7,674,130 amino acids (AA) including 783,158 Xs (10.2%). To place this apparent loss of AA data in perspective, the 52,841 ORFs generated from the 454 data alone, contained 5,579,487AA. Therefore, despite the ~10% loss of AA data in the consensus approach, we were still able to generate 1,311,485 more AA data, an increase of approximately one third, using the combined platform approach.

**Figure 1:**
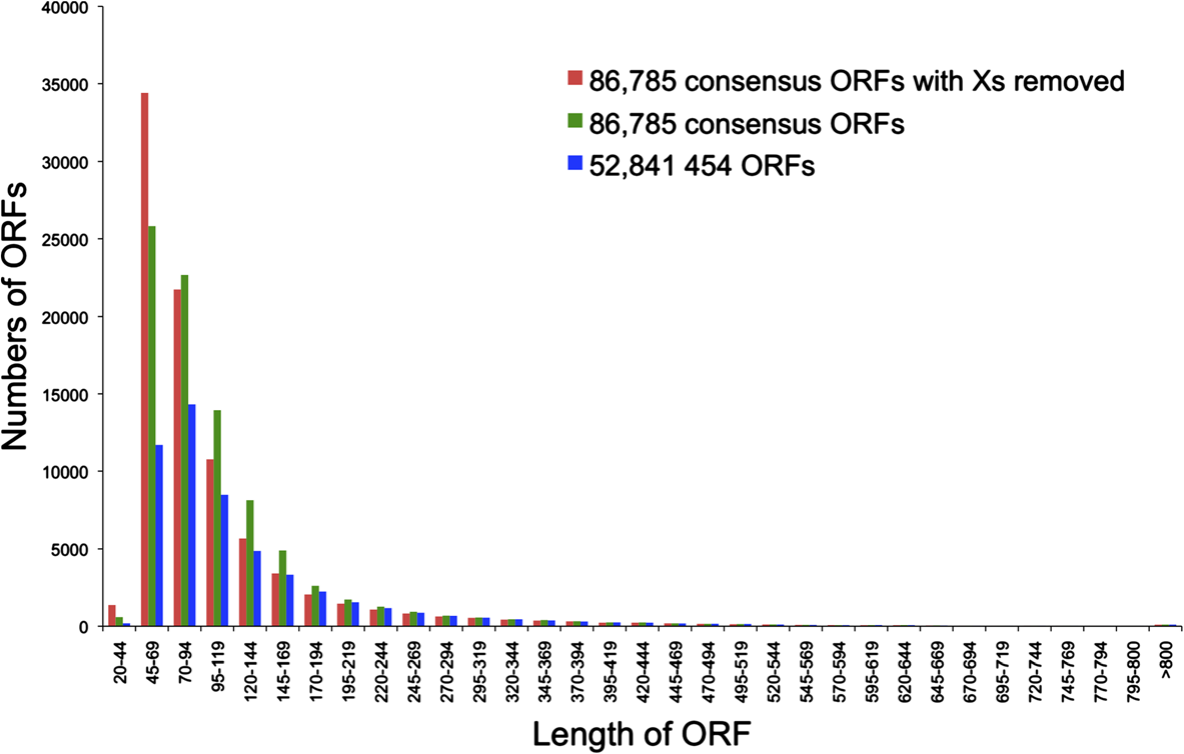
**Length distribution of ORFs generated using 454/Illumina consensus sequences (green bars) and 454 data exclusively (blue bars).** Red bars show distribution for consensus ORFs with unspecified or unknown amino acids (X) removed (see text).

Lengths of ORFs generated using 454/Illumina consensus sequences and 454 data exclusively, showed in general, similar distributions (Figure 1). A noticeable difference, however, was an increase in the number of shorter ORFs (20AA – 169AA) for the consensus data. The number of shorter reads for the consensus data could be even higher as some consensus sequence ORFs contained X homopolymers at their 3′ end that might have masked a stop codon, which may in turn have erroneously increased the ORF length. Therefore, the length comparison was also performed excluding Xs in the consensus sequence ORFs (Figure 1). Mean ORF lengths were as follows: 454 only = 105.6AA, consensus = 88.4AA, consensus (Xs removed) = 79.4AA. The higher mean for the 454 ORFs most likely reflects the exclusion of singletons, which ranged in size from 101 bp to 931 bp (mean = 313 bp).

### Annotation and comparative gene ontology

The ORF annotation was performed by searching the Swiss-Prot database using BLAST2GO [25], and a total of 21,019 consensus ORFs (23.5%) had blast hits with the database, with 19,277 (21.5%) of these receiving annotation with Gene Ontology (GO) terms (see Additional file 4). In comparison, 16,996 454-derived ORFs (32.2%) had blast hits with the Swiss-Prot database, with 15,597 (29.5%) annotated with GO terms (see Additional file 5). Consequently, although the mean ORF length for the consensus data was lower, there was a considerable increase in the number of annotated ORFs obtained (approximately one fifth), highlighting the improvement gained when 454 and Illumina data are combined.

**Figure 2:**
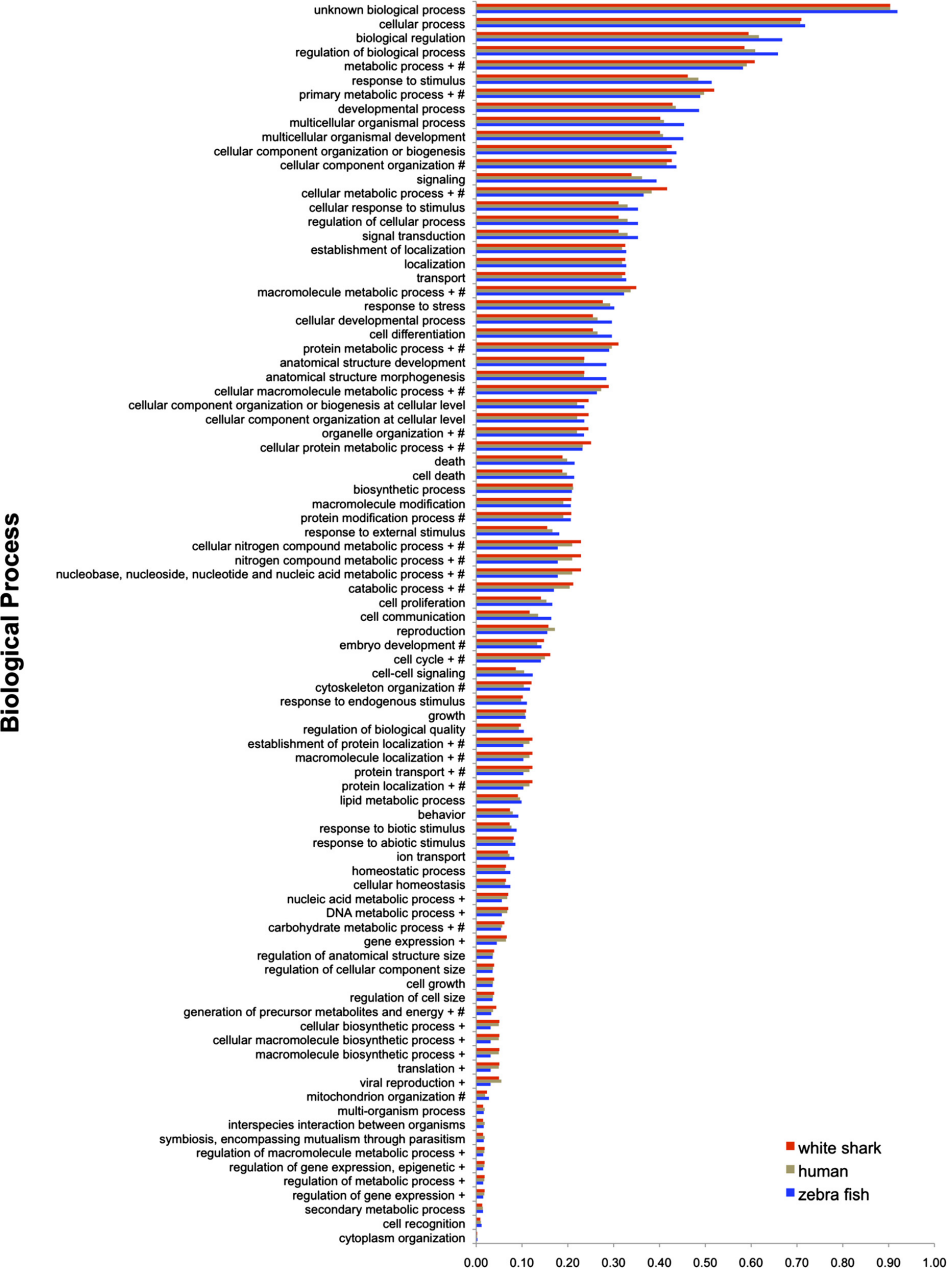
**Proportion of open reading frames assigned to each gene ontology (GO) slim Biological Process term for white shark heart, zebrafish, and human transcriptomes.** The + symbol shows GO terms significantly enriched for the white shark when compared to the zebrafish. The # symbol shows GO terms significantly enriched for the white shark when compared to human.

**Figure 3:**
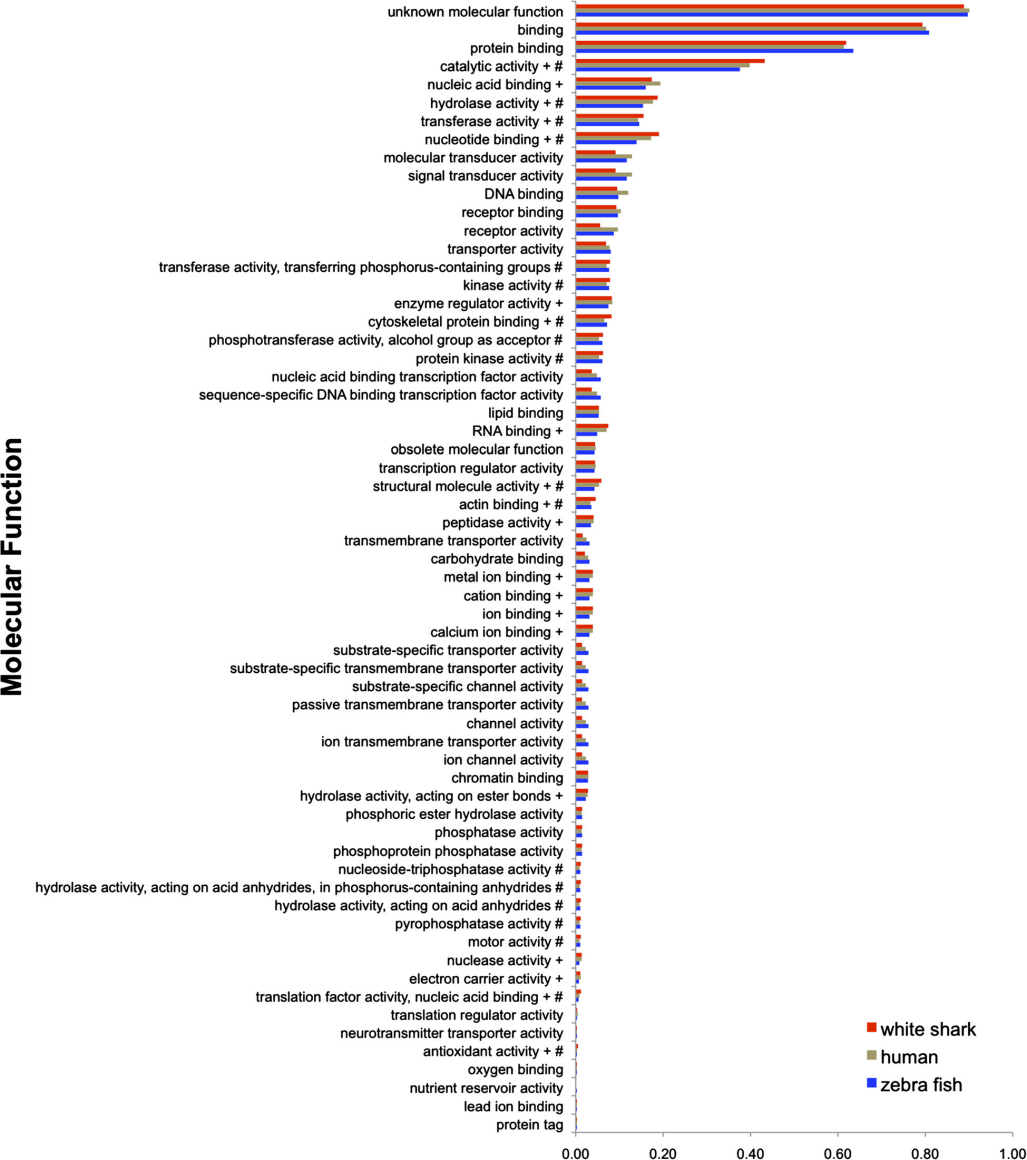
**Proportion of open reading frames assigned to each gene ontology (GO) slim Molecular Function term for white shark heart, zebrafish, and human transcriptomes.** The + symbol shows GO terms significantly enriched for the white shark when compared to the zebrafish. The # symbol shows GO terms significantly enriched for the white shark when compared to human.

**Figure 4:**
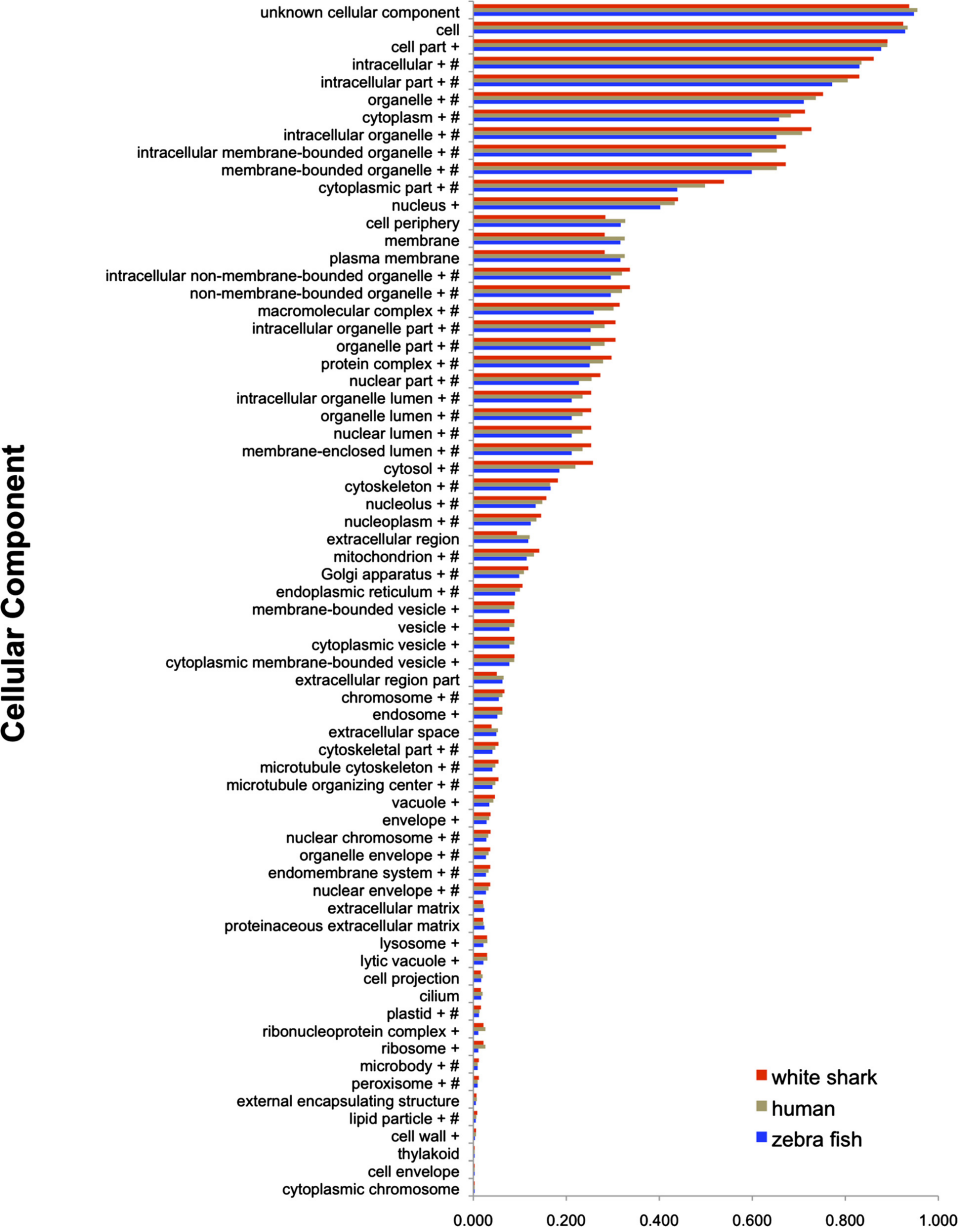
**Proportion of open reading frames assigned to each gene ontology (GO) slim Cellular Component term for white shark heart, zebrafish, and human transcriptomes.** The + symbol shows GO terms significantly enriched for the white shark when compared to the zebrafish. The # symbol shows GO terms significantly enriched for the white shark when compared to human.

The ORFs were also annotated with GO-Slim terms using the generic GO Slim (http://www.geneontology.org/GO_slims/goslim_generic.obo). GO Slim is a reduced version of the full GO that contains a sub-set of more general GO terms and excludes the more fine-grained specific terms. This approach provides a broad overview of the ontology and gene product functions for genomic data. The genome sequence of the zebrafish (*Danio rerio*) is perhaps the most extensively studied of all fishes; consequently, its corresponding transcriptome sequence data should be the most complete for a fish and thus provide an appropriate evolutionary model for comparative characterization of the white shark transcriptome. However, the white shark also possesses certain endothermic capabilities more characteristic of mammals [26,27]; therefore, we compared the proportion of the white shark 19,277 consensus ORFs assigned to each GO Slim term to the proportion of zebrafish and human transcripts assigned to each GO Slim term (Figure 2, 3, 4). Distributions showed generally similar proportions of GO term assignments for each species, suggesting that we obtained a good representation of the heart transcriptome for the white shark. Closer inspection of the GO term proportions in the Biological Process domain (Figure 2) showed that the white shark heart transcriptome had the highest proportion of genes for most of the 18 metabolic process terms, with the zebrafish having the lowest. Fisher tests showed virtually all of these higher white shark proportions to be statistically significant (i.e. 14 of the 18 metabolic terms when compared to the zebrafish and 12 of these terms when compared to human; significance determined using a *FDR* correction of 0.05). Although this comparison is tempered by the fact that the shark transcriptome was derived exclusively from heart tissue and may already be enriched for metabolism relative to the complete transcriptome, it opens the possibility that some aspects of white shark metabolism, at least at the level of gene expression, might be more similar to that of a mammal than to that of an ectothermic teleost. The comparison is tempered because for each term, the Fisher test compares the relative proportion of genes assigned and not assigned the term between a particular species pair (i.e. white shark vs. human and white shark vs. zebrafish). Consequently, the relative proportion of genes assigned a term for the white shark might have been inflated because transcripts derived from other tissue types were absent. Although somewhat speculative, without a complete white shark transcriptome, this apparent higher gene proportion in metabolic process terms compared to zebrafish might be explained partly by the fact that the white shark is not a true poikilotherm. The white shark is among a very small group of fishes that have the ability to physiologically regulate their body temperature and maintain a substantially higher temperature than ambient seawater [26,27], which in turn is associated with elevated metabolic rates and aerobic and anaerobic capacities [28].

For the Molecular Function domain (Figure 3), comparison to the zebrafish showed 20 (32%) GO terms to be significantly enriched (i.e. had a significantly higher proportion of ORFs assigned) in the white shark, whereas comparison to human showed 18 (29%) terms to be enriched. There were 11 terms enriched in the zebrafish comparison that were not enriched in the human comparison. In general, these enriched terms described ion/nucleic acid/RNA binding, and enzyme/peptidase/nuclease/hydrolase/electron carrier activity. In turn, there were nine terms enriched in the human comparison that were not enriched in the comparison to zebrafish. In general, these terms described pyrophosphatase/phosphotransferase/hydrolase/nucleoside/transferase/kinase activity. While many of these enzymatic terms are likely involved in metabolic processes, two terms for the zebrafish comparison are perhaps particularly noteworthy: electron carrier and peptidase activity. Enrichment in these may again reflect the endothermic nature of the white shark. For example, electron carrier term enrichment suggests elevated oxidative metabolism, which is consistent with the increased energetic needs of an endothermic physiology, a continuous swimming lifestyle (required to obtain sufficient ventilation and hydrostatic lift) and the very long distance migratory capability of white sharks [29,30]. Enrichment for the peptidase activity term suggests increased digestive rates in white sharks, consistent with previous hypotheses of this capability based on the elevated temperatures observed in the stomach and other viscera of white sharks [27].

The Cellular Component domain (Figure 4) describes where a gene product is active; and a notably large number of GO terms in this domain were enriched for the white shark: 56% were enriched compared to human and 77% were enriched compared to zebrafish. This unexpectedly large difference in GO term enrichment in the white shark - zebrafish comparison compared to the white shark - human comparison also hints at the possibility that a component of the white shark transcriptome may be more similar to human than zebrafish. Similarities between another chondrichthyian and humans were also apparent in the genome sequence comparisons of Venkatesh et al. [4,31], in which the elephant shark (a chimaera; subclass Euchondrocephali) surprisingly shared a higher degree of gene synteny and more conserved non-coding elements (CNEs) with humans than with either the zebrafish or puffer fish (*Fugu rubripes*).

### Microsatellites

**Figure 5:**
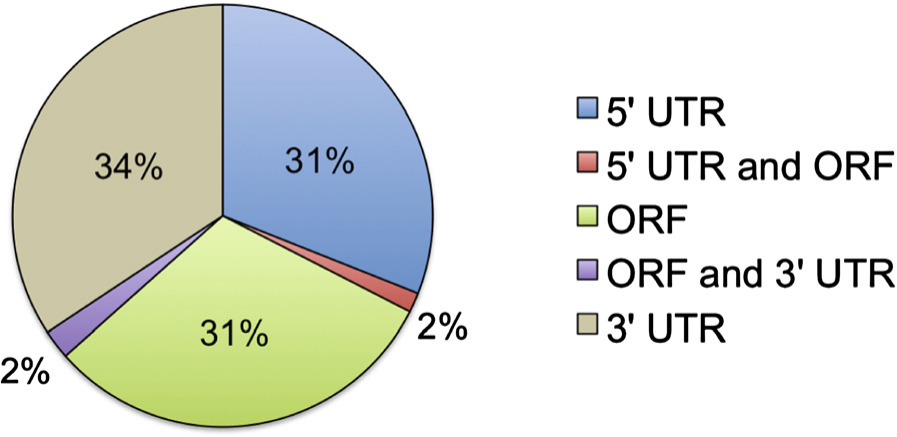
**The proportion of white shark microstatellites within open reading frames (ORF) and untranslated regions (UTR).** Red and purple sections show the proportions for a small number of microsatellites that straddled (i) the 5’ end of the ORF and 5’ UTR and (ii) the 3’ end of the ORF and 3’ UTR.

Roche 454 sequencing has become an effective alternative to established protocols for the isolation of microsatellite markers [32-34], and this approach is increasingly being used to develop such markers for teleost fishes of economic and conservation interest [35,36]. The use of this technology is in its infancy for sharks, with three reports thus far [37-39], all of which were based on 454 genome shotgun sequencing. Here, we provide the results of the first transcriptome based discovery of microsatellite markers and their distributional characteristics in an elasmobranch. Of the 141,626 contigs derived from the 454 white shark data, 6,555 (4.6%) contained one or more dinucleotide, trinucleotide, or tetranucleotide microsatellites of five perfect repeats or more. In total, we detected 8,404 microsatellites with the following motifs: di = 7,467 (88.9%), tri = 864 (10.3%), tetra = 73 (0.9%). The maximum number of repeats for each motif was: di = 63 (average = 13), tri = 31 (average = 6), tetra = 21 (average = 7). See Additional file 6 for a description of the microsatellites. Of the 6,555 contigs containing a microsatellite, 854 were singletons with no consensus sequence. Of the remaining 5,701 contigs, 762 (13.4%) lacked an open reading frame and were therefore possibly non-coding transcripts or transcript fragments. The remaining 4,939 contigs (86.6%) contained one or more microstatellites within the ORF, 5′ untranslated region, and 3′ untranslated region. The proportion of microstatellites within the ORFs and untranslated regions (UTRs) was approximately equal (ORF = 31%, 5′UTR = 31%, 3′UTR = 34%) (Figure 5). A small proportion of microstatellites (4%) straddled the ORF and UTRs.

**Figure 6:**
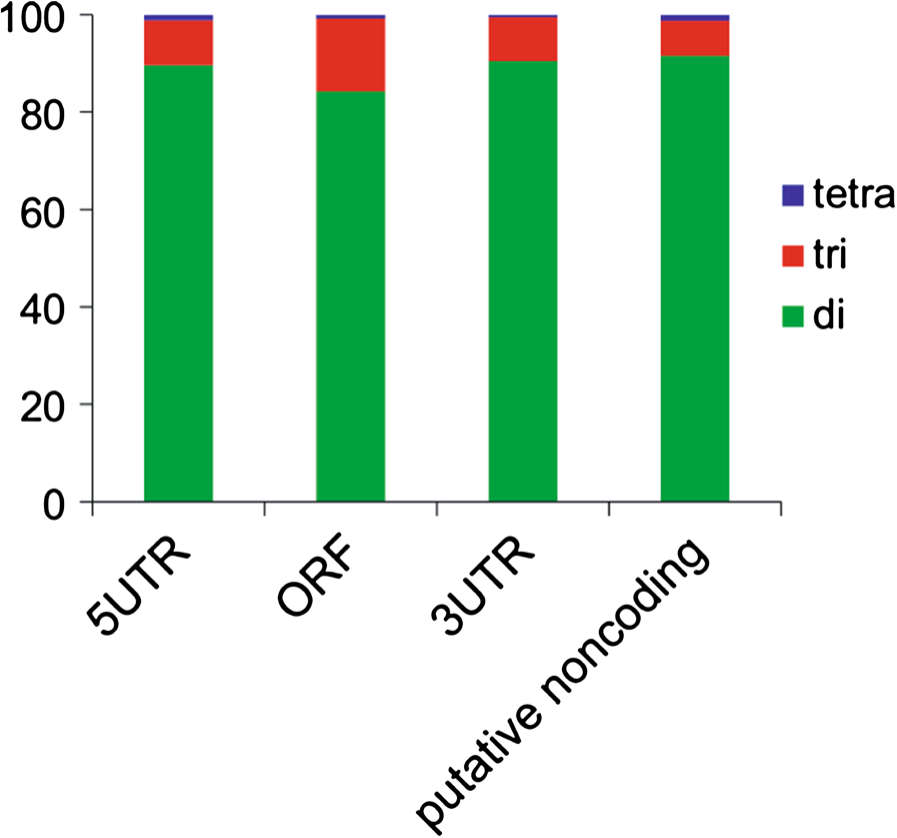
The proportion of white shark di, tri, and tetranucleotide microsatellite repeat motifs within the ORFs, 5’ and 3’ UTRs, and putative non-coding transcripts.

The proportion of di, tri, and tetranucleotide repeat motifs within the ORFs, 5′ and 3′ UTRs, and putative non-coding transcripts are shown in Figure 6. The vast majority of motifs in all transcript types and regions were dinucleotides (ORF = 84%, 5′ UTR = 90%, 3′ UTR = 91%, and putative non-coding transcript = 92%). The only published microsatellites to date from white sharks are also dinucleotides, and were isolated using total genomic DNA and conventional enrichment protocols [40]. A majority of dinucleotide repeat microsatellite motifs were also found in the three shark species (all ectotherms in the order Carcharhiniformes) subject to whole genome 454 sequence analysis [37-39], hinting that a high frequency of dinucleotides may be a general feature of shark genomes. In the white shark, the frequency of repeat motifs within annotated ORFs showed a similar strong bias for dinucleotides (di = 68.8%, tri = 29.5%, tetra = 1.7%). Our finding that dinucleotides were the most frequent repeat motif in white shark transcripts irrespective of transcript region is typical for a wide range of taxonomic groups including other vertebrates [41]. However, when Toth *et al*. [41] only considered exons, trinucleotide repeats were found to be the most frequent. This finding contrasts sharply with that for the white shark where ORFs were strongly dominated by dinucleotide repeats. Another fish, the teleost *F. rubripes*, is a partial exception to the trend of high frequency of trinucleotides in vertebrate coding regions. For example, when repeat motifs of one through eight within ORFs were examined in *Fugu rubripes*, dinucleotides occurred in almost equal proportions to trinucleotides (di = 33.8%, tri = 31.7%) [42]. While the teleost proportions appear to be the most similar to the white shark (perhaps due to a closer evolutionary relationship relative to other vertebrates in this comparison), the white shark remains the most distinctive due to its high proportion of dinucleotides relative to trinucleotides within ORFs.

Expansion of trinucleotide repeats within ORFs has been implicated in human neurodegenerative disorders and some cancers [43-47]. Notably, elasmobranchs allegedly have the lowest incidence of malignant neoplasia (tumors) of any vertebrate group [48], although this claim remains controversial due to a lack of sufficient study [49]. If further studies demonstrate that elasmobranchs do indeed have a lower susceptibility to cancer, the relatively lower proportion of trinucleotide microsatellite repeats within ORFs, as seen here for the white shark, may provide a genetic mechanism hypothesis for further exploration.

There were 1,600 ORFs that contained one or more microsatellite (1,888 microsatellites in total). Of these, 1,331 ORFs contained one or more dinucleotide repeat, 255 ORFs contained one or more trinucleotide repeat, and 14 ORFs contained one or more tetranucleotide repeat. A total of 413 (~ 26%) of these microsatellite-containing ORFs were annotated (motif distribution: di = 284, tri = 122, and tetra = 7). For these ORFs, we investigated whether any of the GO terms assigned to them, appeared in significantly higher proportions (i.e. were relatively enriched) compared to the remainder of the transcriptome’s non-microsatellite containing ORFs. For ORFs containing dinucleotide or tetranucleotide repeats, a Fisher test showed that no GO term was significantly enriched (*FDR* = 0.05). For ORFs containing trinucleotide repeats, however, terms within the Molecular Function domain (nucleic acid/DNA binding and transcription factor/regulator activity) and Cellular Component domain (nucleoplasm) were significantly enriched.

The Molecular Function domain enriched terms described gene products that (i) interacted selectively and non-covalently with nucleic acids, and (ii) interacted selectively and non-covalently with specific DNA sequences in order to modulate transcription. These results suggest that white shark ORFs containing trinucleotide repeats may have regulatory roles involved in the control of transcription (see Additional file 7 for a list of these ORFs).

**Figure 7:**
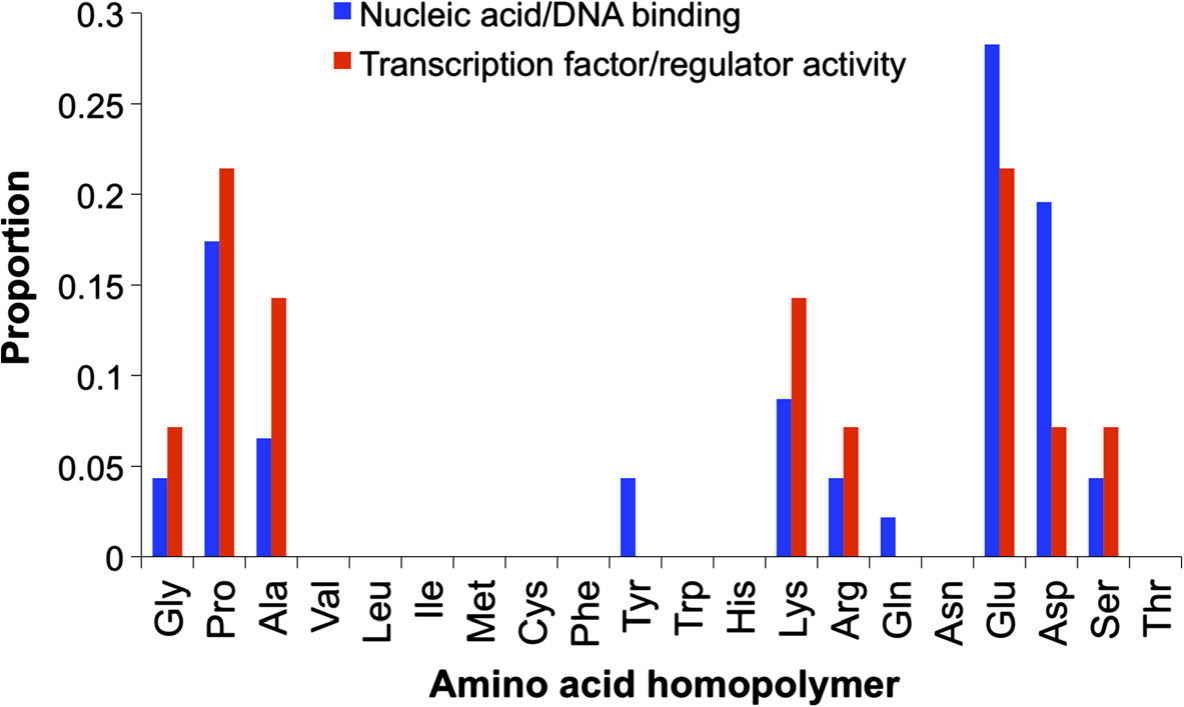
Proportion of amino acid homopolymers within ORFs for (i) the enriched nucleic acid/DNA binding GO term, (ii) the enriched transcription factor/regulator activity GO term.

Previous studies have shown that certain types of trinucleotide repeat, coding for specific amino acid homopolymers, have specific functions. For example, poly-glutamic acid homopolymers are common in nuclear localization signal proteins [50] and have been implicated in transcription activation/de-activation [41,51-53], whereas proline homopolymers may provide a domain for DNA binding and affect protein-protein interactions [51,52]. In general, the white shark was concordant with these findings, as the most frequent amino acid homopolymers within ORFs for the enriched nucleic acid/DNA binding GO term were poly-glutamic acid (28.3%), poly-aspartic acid (19.6%), and poly-proline (17.4%) (Figure 7). There was a similar pattern for the enriched transcription factor/regulator activity GO term with poly-glutamic acid (21.4%) and poly-proline (21.4%) being the most frequent (Figure 7). The white shark was distinctive however, in that there was a large proportion of poly-aspartic acid homopolymers within ORFs with regulatory roles (i.e. nucleic acid/DNA binding).

Finally, the large pool of microsatellites discovered here provides the potential to greatly expand the limited microsatellite marker resources available for this vulnerable species. To this end and as part of a separate study, we are developing microsatellite PCR primers on a global set of white shark fin tissue samples. To date, we have tested 35 loci (mostly dinucleotide and trinucleotide repeats). Of these, 14 are scorable (an individual can be genotyped), suggesting good prospects for the development of additional loci (A. Bernard, VPR, MJS, MSS; data not shown).

### Positive selection

We searched the white shark transcriptome for genes showing signs of positive selection by comparing it to embryo transcriptomes of two additional elasmobranch species: *Scyliorhinus canicular* (cat shark) and *Leucoraja erinacea* (little skate). For each of the three species, we tested each species’ lineage for positive selection using the branch-site test as implemented in PAML [54]. Before correction for multiple testing, there were ten, three, and five genes on the white shark, cat shark, and skate lineages respectively that had significant results for positive selection (Additional file 8 shows results for white shark). After correction (*FDR* = 0.05), four white shark genes remained significant: UN031816 (TIP41-like protein), UN034361 (mediator of RNA polymerase II transcription subunit 20), UN050025 (protein MIS12 homolog), and UN034642 (uncharacterized protein C12orf12 homolog). None of the cat shark or skate genes remained significant after correction for multiple testing.

In yeast, TIP41 indirectly regulates cell growth by regulating SIT4 (serine/threonine-protein phosphatase 2A) activity [55]. More specifically, when nutrients such as nitrogen or carbon are abundant, the rapamycin-sensitive TOR signaling pathway promotes binding of the inhibitor protein TAP42 to SIT4 thereby inhibiting its activity. However, when nutrients are low, TOR does not promote binding of TAP42 and the inhibitor disassociates from SIT4 and TIP41 binds to the inhibitor, which in turn permits SIT4 activity. The regulatory role of protein phosphatases is debated in the literature, and they may function to both up and down regulate cell growth [56]. Furthermore, given the important role of these enzymes in cell growth, they have been actively studied by cancer researchers, with some studies suggesting that they might possess tumor suppressive capabilities [56]. However, other studies have emphasized their requirement for active cell growth and survival [56]. Nevertheless, the finding here of positive selection for a white shark gene involved in their regulation warrants further investigation given (i) an apparent low incidence of malignant tumors reported for elasmobranchs [48], (ii) the high levels of nitrogen (urea) in shark tissue, and (iii) the unique ability of elasmobranchs when compared to higher vertebrates to regenerate kidney tissue [57,58].

Mediator of RNA polymerase II transcription subunit 20 is a component of the Mediator complex. This large multi protein complex, which is conserved among eukaryotes, binds RNA polymerase II and regulates transcription of class II genes [59-61]. In addition to controlling cell growth, the TOR signaling pathway has also been implicated in the regulation of transcription. For example, in yeast, TOR limits transcription when nitrogen levels are low [62]. Perhaps the elevated level of urea in shark tissue is a factor contributing to positive selection for the Mediator subunit 20.

Using Blast2GO, we were able to assign GO terms to three of the genes showing signs of positive selection (UN034361, UN031816, and UN050025) (14 terms in total) (Additional file 8). Montoya-Burgos [63] compared GO terms enriched for genes under positive selection among two teleosts and six eutherian mammals (including humans). Comparison of these results to the GO terms for the white shark genes under positive selection is shown in Table 1. Similarities were regulation of transcription for the Biological Process domain and protein binding for the Molecular Function domain. A notable difference, however, was the complete absence of Biological Process response terms for the white shark (and also the cat shark and skate). In contrast, multiple response terms were shared between the teleosts and mammals (e.g. response to stimulus, stress, and wounding; defense response; and immune response). Furthermore, numerous studies involving a variety of additional teleosts have reported detection of positive selection for genes involved in stress and immune system response [64-68]. Elasmobranchs are the most primitive jawed vertebrate to possess an adaptive immune system based on immunoglobulins, T cell receptors, and major histocompatibility complex molecules (Ig/TCR/MHC). However, this system is genetically distinct from the higher vertebrates [69,70] and has a restricted antibody response when compared to teleosts and mammals [71,72]. The lack of positive selection for genes involved in immune and defense response reported here for three elasmobranch species might in part reflect this elasmobranch immune system distinctiveness.

**Table 1:** Comparison of GO terms for genes under positive selection for the white shark, two teleosts, and six mammals

**Biological process**	**Biological process**
**(white shark)**	**(shared between teleosts and mammals)**
Biological regulation	Response to stimulus
Regulation of transcription from RNA polymerase II promoter	Defense response
Cell communication	Response to stress* or wounding**
Chromosome segregation	Immune system process, Immune response
Kinetochore assembly	Signal transduction
	Regulation of transcription DNA-dependent
	Ion transport
**Molecular function**	**Molecular function**
**(white shark)**	**(shared between teleosts and mammals)**
Protein binding	DNA binding, or mismatch DNA binding
RNA polymerase II transcription mediator activity	Protein binding**, chemokine receptor binding**, interleukin binding**, interleukin-1 receptor binding*
DNA-directed RNA polymerase activity	Metal ion binding

GO terms shared between teleosts and mammals are from Montoya-Burgos [63]. A single asterisk shows terms found in teleosts. Two asterisks shows terms found in mammals.

## Conclusions

Utilizing an approach that combined Roche 454 and Illumina sequencing technologies, we assembled and characterized the first white shark transcriptome. This combined approach yielded a considerable improvement over Roche 454 technology alone, generating 21,019 annotated transcripts. The white shark transcriptome is a valuable resource that adds to the currently nascent field of cartilaginous fish genomics and provides a reference for characterization of genomic datasets from other elasmobranchs, which we anticipate will emerge with increasing frequency. This resource also provides the first large-scale view of the gene content of a major marine apex predator that displays a collection of remarkable physical, physiological, and behavioral properties. Of particular interest is the observation that the proportion of annotated transcripts involved in metabolic processes was more similar between the white shark and humans than between the white shark and a teleost, a finding consistent with those of Venkatesh *et al*. [4,31] who found genomic non-coding elements and the relative position of genes to be more similar between another cartilaginous fish (the elephant shark) and humans than between the elephant shark and a teleost. We also compared the white shark transcriptome to other available elasmobranch sequences, for signatures of positive selection and identified several genes of putative adaptive significance on the white shark lineage. The transcriptome resource also provides a large set of new microsatellites that will be immediately useful as markers in studies of population structure, dispersal dynamics, genetic diversity, and mating system biology to further the conservation and management of this vulnerable species.

## Methods

### Tissue collection

The white shark is protected by many countries, including the US, and is also a CITES Appendix II listed species [73]. Consequently, obtaining white shark tissue is extremely difficult. However, we were able to obtain tissue from a juvenile white shark illegally landed by an independent fisher off the Delaware, USA coast in 2007. The shark was confiscated from the fisher by the US National Oceanic and Atmospheric Administration Office for Law Enforcement. The heart was collected during a subsequent necropsy of the shark conducted by the National Oceanic and Atmospheric Administration for scientific data collection, and provided to us by this agency for further analysis. The heart was kept frozen at -80°C until sub-sampled for RNA isolation.

### cDNA library construction and Roche 454/Illumina sequencing

Total RNA was isolated by homogenization of heart tissue in TRIzol (Invitogen, Carlsbad, USA) followed by phenol chloroform extraction. Full-length cDNA was synthesized using two sets of oligo dT primers in a two step procedure and single-stranded cDNA was used for hybridization instead of double-stranded [74]. After hybridization, reassociated ds-cDNA was separated from ss-cDNA (normalized cDNA) by hydroxyapatite chromatography. Normalized cDNA was re-amplified using an oligo dT specific primer (L4N). cDNA was sequenced using a single run on the Roche 454 GS FLX platform and a single lane of Illumina HiSeq 2000 (100 bp reads, single end).

### Sequence assembly and annotation

Roche 454 adaptor sequences were removed using LUCY [75] and the script SeqClean (http://compbio.dfci.harvard.edu/tgi/software). SeqClean was also used to remove reads containing low complexity sequence, reads shorter than 100bp, and to clip low quality read ends (ends rich in undetermined bases). 454 reads were assembled into contigs *de-novo* using iAssembler v1.3 [76]. Contigs were searched for di, tri, and tetra microsatellites of five repeats or more using Phobos v3.3.12 as implemented in Geneious v5.5.3 [77]. Illumina HiSeq reads were aligned to 454 contigs using the program Burrows-Wheeler Aligner (BWA) [78] and consensus sequences built using the pileup format as implemented in SAMtools [79].

Roche 454/Illumina consensus sequences were searched for open reading frames (ORFs) of 20 amino acids or longer (including the start codon for methionine, but omitting unspecified or unknown amino acids [coded as X]) using the script longorf.pl (available at http://search.cpan.org/~cjfields/BioPerl-1.6.901/examples/longorf.pl). Non-singleton 454 contigs lacking Illumina read coverage were also searched for ORFs using the longorf.pl procedure. Annotation for ORFs was obtained using Blast2GO v.2.5.0 [25]. Amino acids were searched against the UniProtKB/Swiss-Prot database using an *E* value cut-off = 1e-6 (retaining best 20 hits), with a minimum amino acid alignment length cut-off (high-scoring segment pair length) of 33. Blast2GO was also used to assign GO terms.

For purposes of quantitative evaluation of our combined 454/Illumina sequencing platform approach, we also processed the 454 data without combining it with Illumina data. After singletons were removed, the contigs were searched for ORFs and annotated using the same procedure as for the combined data.

Blast2GO was also used to annotate ORFs and assign GO terms for the zebrafish and human transcriptomes (same procedure as above) to provide comparison to the white shark. Transcriptomes for zebrafish and human were obtained from Ensembl (Danio_rerio.Zv9.66.cdna.all.fa, Homo_sapiens.GRCh37.67.cdna.all.fa). Note: the Ensemble cdna.all files contain “the super set of all transcripts resulting from Ensemble known, novel and pseudo gene predictions” (see the associated readme file for a complete description). Relative enrichment of GO terms for white shark when compared to zebrafish and human (separate comparisons) was assessed using a Fisher exact test. The test was performed using the Gossip statistical package [80] implemented within Blast2GO. The false discovery rate (FDR) procedure of Benjamini and Hochberg [81] was used to correct for multiple hypothesis testing (*FDR* = 0.05). We did not test for underrepresentation (lower proportion of terms) as the white shark transcriptome was obtained from a single tissue type and may therefore not represent complete genomic expression.

### Branch-site test of positive selection

In order to detect genes under positive selection using the branch-site test [82], we obtained embryo transcriptome data for two additional elasmobranch species: *Scyliorhinus canicula* (smallspotted cat shark) and *Leucoraja erinacea* (little skate). The data were downloaded from the Gene Expression Omnibus database at NCBI (accession number GSE26235). Transcripts were searched for open reading frames of 20 amino acids or longer using the same procedure as for the white shark. For each of the three elasmobranch species (*C. carcharias*, *S. canicular* and *L. erinacea*), and their putative homologous loci (procedure described below), we tested genes in each species’ lineage for positive selection using the branch-site test as implemented in codeml in PAML (Phylogenetic Analysis by Maximum Likelihood) version 4.4 [54]. The test was performed on homologous core genes (those genes shared among all three species). Homologous genes were delineated using the MCL algorithm [83] as implemented in the MCLBLASTLINE pipeline (available at http://micans.org/mcl). The pipeline uses Markov clustering (MCL) to assign genes to homologous clusters based on a BLASTp search between all species pairs of protein sequences using an *E* value cut-off of 1e-5. The MCL algorithm was implemented using an inflation parameter of 1.2. Only single copy core genes were used (i.e. clusters containing paralogs were excluded). The nucleotide sequences corresponding to each set of homologous core genes were aligned using Probalign [84]. Alignment columns with a posterior probability <0.6 were removed, and alignments with >50% of the sites removed were discarded from the analysis. Using each of the alignments and the three elasmobranch species tree topology, positive selection was assessed for each lineage by performing likelihood ratio tests. We compared two branch-site models: (i) a null model that does not allow positive selection (model M1a) and (ii) an alternative model that allows positive selection (model A). *P* values were calculated under the assumption that the likelihood ratio follows a chi-square distribution with one degree of freedom [82]. Multiple testing adjustment was performed using a false discovery rate approach [85] (significance level = 0.05).

### Availability of supporting data

The 454 and Illumina derived short read files are available at the NCBI Sequence Read Archive (SRA) under the study accession number SRP016555.

## Competing interests

The authors declare that they have no competing interests.

## Authors’ contributions

MSS and MJS conceived, developed, and supervised the project. VPR and HS conducted the bioinformatics analyses. VPR drafted the manuscript with contributions from MSS and HS. All authors contributed to editing the manuscript. All authors read and approved the final manuscript.

## Supplementary Material

Additional file 1 (454 contigs). Click here for file

Additional file 2 (454/Illumina consensus nucleotide sequences). Click here for file

Additional file 3 (454/Illumina consensus ORFs [amino acid sequences]). Click here for file

Additional file 4 (annotation of 454/Illumina consensus ORFs). Click here for file

Additional file 5 (annotation of 454 ORFs). Click here for file

Additional file 6 (microsatellite characteristics). Click here for file

Additional file 7 (annotated ORFs containing trinucleotide microsatellite repeats associated with enriched GO terms). Click here for file

Additional file 8 (ORFs significant for positive selection). Click here for file
